# Increasing Thyroid Cancer Rate and the Extent of Thyroid Surgery in Korea

**DOI:** 10.1371/journal.pone.0113464

**Published:** 2014-12-03

**Authors:** Myung-Whun Sung, Bumjung Park, Soo-Youn An, J. Hun Hah, Young Ho Jung, Hyo Geun Choi

**Affiliations:** 1 Department of Otorhinolaryngology-Head & Neck Surgery, Seoul National University College of Medicine, Seoul, Korea; 2 Department of Otorhinolaryngology-Head & Neck Surgery, Hallym University Sacred Heart Hospital, Anyang, Korea; 3 Department of Otorhinolaryngology-Head & Neck Surgery, Thyroid/Head & Neck Cancer Center of the Dongnam Institute of Radiological & Medical Sciences (DIRAMS), Busan, Korea; 4 Department of Otolaryngology-Head and Neck Surgery, Seoul National University Boramae Hospital, Seoul, Korea; Uppsala University, Sweden

## Abstract

**Background:**

It is evident that the rate of thyroid cancer is increasing throughout the world. One reason is increased detection of preclinical small cancers. However, it is not clear whether the increase in thyroid cancer rate is reducing the extent of thyroid surgeries. The purpose of this study was to evaluate the thyroid cancer rate and analyze recent changes in the extent of thyroid cancer surgeries in Korea.

**Methods:**

An observational study was conducted using data from Korea’s Health Insurance Review and Assessment Service (HIRAS) for thyroidectomy with/without neck dissection, with 228,051 registered patients between 2007 and 2011. Data were categorized by the extent of surgery: unilateral thyroidectomy without neck dissection (*UT*), bilateral thyroidectomy or radical thyroidectomy without neck dissection (*TT*), any thyroidectomy with unilateral selective neck dissection (*SND*), any thyroidectomy with unilateral modified radical neck dissection (*MRND*), any thyroidectomy with unilateral radical neck dissection (*RND*), and any thyroidectomy with bilateral neck dissection (*BND*). Annual rate difference for each surgery was analyzed with a linear by linear association.

**Results:**

The absolute numbers of total thyroid surgeries (*UT+TT+SND+MRND+RND+BND*) were increased from 28539 to 61481. The proportion of patients who underwent only thyroidectomy without neck dissection (*UT+TT*) decreased from 67.30% to 60.50%, whereas the proportion of patients who underwent neck dissection (*SND+MRND+RND+BND*) increased from 32.70% to 39.50% during the 5-year study period.

**Conclusion:**

Despite the increase in rate of thyroid cancer due to earlier detection, increased rate of neck dissection was noted.

## Introduction

The rate of thyroid cancer is exploding all over the world. The incidence of thyroid cancer has increased from 2.4% to 9.4% annually in the last three decades [1]. There are two explanations for this increase. One is that early detection of small cancers at a preclinical stage has recently increased because of screening and development of diagnostic tools [2]. The other is that thyroid cancer rate is truly increasing because of radiation, diet, lifestyle, and environmental changes [3], [4]. Many other reports have demonstrated that not only microcarcinomas (≤1 cm) but also large thyroid cancers (>4 cm) are increasing [5]–[8].

However, there is a lack of data on changes in the rate and extent of lymph node (LN) metastasis of thyroid cancer as it relates to the increase of thyroid cancer rate [9], [10]. Xiang J and colleagues demonstrated that thyroid cancer was discovered at an earlier stage with an increasing rate. Not only the tumor size decreased, but also the rate of extrathyroidal extension decreased. The rate of central cervical LN metastasis decreased from 66.2% (99/128) in 1996 to 52.6% (301/572) in 2006. During this period, the number of thyroid cancer increased 3.9-fold in eastern China [10]. Miki and colleagues reported that thyroid cancer patients detected earlier by mass screening had less LN metastasis than those who visited outpatient clinics in Japan. The tumor size in the mass-screening group (14±6 mm) was significantly smaller than in patients presenting at an outpatient clinic (19±13 mm), and the rate of nodal metastases in the mass-screening group (38%) was significantly lower than in the outpatient group (68%) [11]. These results support that increasing rate of thyroid cancer is related to increased early cancer detection with less frequent LN metastasis.

The rate of thyroid cancer in Korea is among the highest in the world [12], and thyroid cancer rates continue to increase. Thyroid cancer is the most common cancer in Korea (the 1^st^ in women and the 6^th^ in men). The age-standardized rate (ASR) per 100,000 increased from 38.1 in 2007 to 61.9 in 2011. The ASR per 100,000 in women and men increased from 64.8 and 11.6 to 102.8 and 21.6 between 2007 and 2011, respectively. Ninety-five percent of thyroid cancer was papillary thyroid cancer [13]. Because the prevalence of thyroid cancer in Korea has been increasing rapidly in recent years, changes in the rate and extent of thyroid cancer surgery are expected.

In this study, we analyzed chronological changes of the extent of thyroid cancer surgery with the expectation that the rate of patients undergoing thyroidectomy without neck dissection would increase, while the rate of patients who underwent neck dissection would decrease.

## Materials and Methods

### Study Population and Data Collection

This study was approved by the institutional review board of Seoul National University Hospital (SNUH IRB No. E-1208-113-422).

This study included all of Korea without exceptions. We acquired relevant data for the years 2007 to 2011 for the entire Korean population, which is about 50 million people, from the National Health Insurance Corporation. The population data were counted as of the last day of each year. Because all Korean people are given a 13-digit resident registration number from birth to death, exact population statistics can be determined from the database. It is mandatory for all Koreans to be enrolled in the national health insurance scheme. We analyzed the population data for age intervals from 0 to 95 years. People over 95 years of age were grouped into one age group.

Health Insurance Review & Assessment Service (HIRAS) data for thyroidectomy, neck dissection, and related surgeries from January 1, 2007 to December 31, 2011 were analyzed; there were 228,051 cases. HIRAS is the government organization of Korea and Data was collected and sorted by the expert statisticians of the HIRAS who are not involved in this study. All cases were diagnosed as malignant neoplasm of the thyroid gland (C73) by ICD-10 classification and underwent thyroidectomy with or without neck dissection. The data was categorized into six groups: unilateral thyroidectomy without neck dissection (*UT*), bilateral thyroidectomy or radical thyroidectomy without neck dissection (*TT*), any thyroidectomy (*UT* or *TT*) with unilateral selective neck dissection (*SND*), any thyroidectomy with unilateral modified radical neck dissection (*MRND*), any thyroidectomy with unilateral radical neck dissection (*RND*), and any thyroidectomy with bilateral neck dissection (*BND*). “*SUM*” was defined as the summation of all the surgeries listed above (Table 1). Neck dissection only cases were not included in this study. All hospitals and clinics should be listed in the HIRAS database, and all use the same surgery codes because of the single national medical insurance system.

**Table 1:** Types of thyroid cancer surgeries.

Type	Code	Surgery
UT	P4551, P4553	Unilateral thyroidectomy without neck dissection
TT	P4552, P4554, P4561	Bilateral thyroidectomy or radical thyroidectomy without neck dissection
SND	P2114, P2117	Any thyroidectomy with unilateral selective neck dissection
MRND	P2113, P2116	Any thyroidectomy with unilateral modified radical neck dissection
RND	P2112, P2115	Any thyroidectomy with unilateral radical neck dissection
BND	P2118, P2119	Any thyroidectomy with bilateral neck dissection
SUM		Summation of all the surgeries listed above

### Study Variables

There could be some different strategies for the extent of thyroid cancer surgery between surgeons. However, we assumed that most surgeons performed neck dissections only when LN metastasis was suspected clinically or the size of primary tumors was large (T stage 3–4) according to the guidelines of Korean thyroid association which is similar with the American Thyroid Association (ATA) guidelines. ATA recommends functional compartmental en-bloc neck dissection when nodal disease is evident clinically, on preoperative US and nodal fine needle aspiration or thyroglobulin measurement, or at the time of surgery. In addition, prophylactic neck dissection is recommended in the case of T stage 3–4 tumors [14]. When neck metastasis is suspected, neck dissection is usually performed according to the range of suspected LN metastasis. This guideline was also recommended for thyroid cancer surgeries in Korea.

The extent of thyroid cancer surgery was increasingly radical in the order *UT, TT, SND, MRND, RND*, and *BND*. Almost all surgeons code the surgeries correctly according to the guidelines, and this is supervised by HIRAS. There could be minor differences among surgeons when coding surgeries, which could create a bias in this study. However, this study analyzed yearly changes of surgery rate in the whole country rather than individual differences. Because data for this study came from all of the hospitals and clinics in Korea, any bias from potential confounders, such as indication differences between individual surgeons or institutions, should be minimized [15].

In Korea, cancer registration is very important and accurate because health care costs are associated with 95% discounts by National Health Insurance. Because all Korean hospitals and clinics always use the 13-digit resident registration number to register individual patients for the medical insurance system, the risk of overlapping medical records is practically nil, even if a patient moves from one place to another for further treatment.

The number of foreigners registered with the National Health Insurance of Korea was less than 1% of the Korean population during the 2007 to 2011 time period. Thus, we assume that our data reflect the ethnic characteristics of the Korean people [16].

There was no *BND* code in 2007. In that year, despite bilateral neck dissection, it was coded as *MRND* or *RND* according to the range of neck dissection.

### Statistical Analysis

The annual rate differences for the surgeries were assessed using a linear by linear association. The mean age and gender differences between surgeries were calculated with an independent-sample T test. The results were analyzed statistically using SPSS software (ver. 20.0; SPSS Inc., Chicago, IL, USA).

## Results

### Study Population

A total of 228,051 patients (38,246 male and 189,805 female) in Korea who were diagnosed with malignant neoplasm of the thyroid gland (C73) and underwent thyroidectomy with/without neck dissection from January 1, 2007 to December 31, 2011 made up the study cohort. The male-to-female ratio was 1∶4.9. The mean age of patients was 47.83±11.68 years (47.36±11.93 for men and 47.92±11.62 for women).

### Study Outcome

All kinds of thyroid surgery with/without neck dissection (*SUM*) increased 2.17 times from 28,539 cases to 61,481 cases during the 5 years. *SUM* for men increased more rapidly than for women. *SUM* increased 2.55 times and 2.08 times for men and women, respectively, for the 5 years (Table 2). All of these results were statistically significant (*P*<.001).

**Table 2:** The number and proportion of thyroid surgeries.

Type	Year	Male		Female		Total		Proportion (%)†
		Number	Change*	Number	Change	Number	Change	
**UT**								
	2007	341	1	2038	1	2379	1	8.34
	2008	475	1.39	2532	1.24	3007	1.26	7.86
	2009	723	2.12	3620	1.78	4343	1.83	9.14
	2010	961	2.82	4133	2.03	5094	2.14	9.75
	2011	1178	3.45	4912	2.41	6090	2.56	9.91
**TT**								
	2007	2495	1	14334	1	16829	1	58.97
	2008	3448	1.38	18388	1.28	21836	1.30	57.04
	2009	4064	1.63	21298	1.49	25362	1.51	53.38
	2010	4596	1.84	22479	1.57	27075	1.61	51.83
	2011	5384	2.16	25723	1.79	31107	1.85	50.60
**SND**								
	2007	904	1	5260	1	6164	1	21.60
	2008	1383	1.53	7100	1.35	8483	1.38	22.16
	2009	1821	2.01	9531	1.81	11352	1.84	23.89
	2010	2140	2.37	10595	2.01	12735	2.07	24.38
	2011	2722	3.01	12672	2.41	15394	2.50	25.04
**MRND**								
	2007	341	1	1456	1	1797	1	6.30
	2008	332	0.97	1225	0.84	1557	0.87	4.07
	2009	417	1.22	1492	1.02	1909	1.06	4.02
	2010	389	1.14	1321	0.91	1710	0.95	3.27
	2011	400	1.17	1302	0.89	1702	0.94	2.77
**RND**								
	2007	225	1	1145	1	1370	1	4.80
	2008	156	0.69	846	0.74	1002	0.73	2.62
	2009	134	0.55	580	0.51	714	0.52	1.50
	2010	156	0.69	607	0.53	763	0.56	1.46
	2011	161	0.72	710	0.62	871	0.64	1.42
**BND**								
	2007	0		0		0		0
	2008	394	1	2001	1	2395	1	6.26
	2009	590	1.50	3243	1.62	3833	1.60	8.07
	2010	773	1.96	4088	2.04	4861	2.03	9.31
	2011	1143	2.90	5174	2.59	6317	2.63	10.27
**SUM**								
	2007	4306	1	24233	1	28539	1	
	2008	6188	1.44	32092	1.32	38280	1.34	
	2009	7749	1.80	39764	1.64	47513	1.66	
	2010	9015	2.09	43223	1.78	52238	1.83	
	2011	10988	2.55	50493	2.08	61481	2.17	

*Change = Number in each year/Number in 2007. (BND was calculated with respect to 2008.).

† Proportion of each surgery for each year (example: UT prop in 2007 = Number of UT in 2007/Number of all surgeries in 2007).

*UT* increased 2.56 times, from 2,379 cases to 6,090 cases, while *TT* increased only 1.85 times, from 16,829 cases to 31,107 cases during the 5 years. Thus, the proportion of *UT* increased from 8.34% in 2007 to 9.91% in 2011, whereas the proportion of *TT* decreased from 58.97% in 2007 to 50.60% in 2011. The proportion of thyroidectomy without neck dissection (*UT+TT*) decreased from 67.30% in 2007 to 60.50% in 2011 (Table 2). All of these results were statistically significant (*P*<.001).

**Figure 1:**
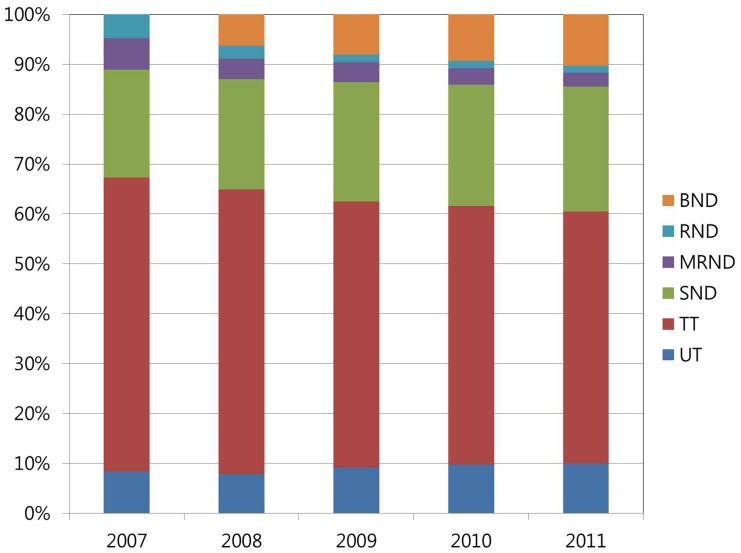
The proportions of thyroid surgeries from 2007 to 2011. The proportion of SND+MRND+RND+BND increased from 32.70% in 2007 to 39.50% in 2011. In contrast, the proportion of UT+TT decreased from 67.30% in 2007 to 60.50% in 2011.

*SND* increased 2.50 times from 6,164 cases to 15,394 cases, while *MRND* decreased 0.94 times from 1797 cases to 1702 cases and RND decreased 0.64 times from 1770 cases to 763 cases in 5 years of study period. *BND* increased 2.63 times from 2,395 cases to 6,317 cases between 2008 and 2011. Neck dissection with any thyroidectomy (*SND+MRND+RND+BND*) increased 2.6 times from 9,331 cases to 24,284 cases. The proportion of neck dissection with any thyroidectomy (*SND+MRND+RND+BND*) also increased from 32.70% in 2007 to 39.50% in 2011 (Table 2) (Fig. 1). All of these results were statistically significant (*P*<.001).

*UT, TT, SND, MRND, RND,* and *BND* increased more rapidly in men than in women from 2007 to 2011. Thus, the male-to-female ratio was 5.63 in 2007 and changed to 4.60 in 2011. All of these results were statistically significant (*P*<.001).

### Other results

We analyzed the mean ages of patients when surgeries were performed. The differences of mean age between surgeries were statistically significant in almost all comparisons. Patients who underwent *UT* (45.47 years) were the youngest, and those undergoing *MRND* (46.81 years) were the next youngest. Other surgeries were performed when patients were around age 48 years. Differences between male and female patients were less than 1 year for all kinds of surgeries. Despite statistical significance, this difference is clinically meaningless (Table 3).

**Table 3:** Mean age of patients undergoing each type of surgery.

Type	Male	Female	P-value*	Total
UT	46.25	45.30	.000	45.47
TT	47.61	48.28	.000	48.17
SND	47.56	48.10	.000	48.01
MRND	46.54	46.88	.308	46.81
RND	47.20	47.69	.326	47.60
BND	46.94	48.54	.000	48.28
SUM	47.36	47.92	.000	47.83

*P-value of independent T-test between male and female patients.

## Discussion

The proportion of neck dissections in thyroid cancer surgery increased annually between 2007 and 2011 in this study. We assumed that the proportion of neck dissections would decrease if thyroid cancers, especially small cancers, are increasing due to the early detection effect. However, the result of this study was the opposite. The proportion of neck dissections as well as the absolute number of neck dissections among thyroid cancer surgeries increased. Neck dissection with thyroidectomy (*SND+MRND+RND+BND*) increased 2.6 times from 9,331 cases to 24,284 cases in this study. This result is in accordance with results in the United States, where the number of neck dissections for thyroid and parathyroid disease increased from 2,822 in 2000 to 5,282 in 2006 [17]. Moreover, the proportion of patients who underwent neck dissection with thyroidectomy (*SND+MRND+RND+BND*) increased from 32.70% in 2007 to 39.50% in 2011. In contrast, the proportion of the patients who underwent only thyroidectomy without neck dissection (*UT+TT*) decreased from 67.30% in 2007 to 60.50% in 2011. This may suggest that the risk of post-operative morbidity due to LN dissection is increased. LN dissection elevates postoperative morbidities, including permanent hypoparathyroidism and unintentional injury to the recurrent laryngeal nerve, spinal accessory nerve dysfunction, and/or chyle leakage [18], [19] compared with thyroidectomy alone [20]. It is hard to demonstrate that the increased proportion of neck dissection is directly associated to increased morbidity of surgery, because radical surgery such as RND and MRND decreased and there were no data about specific morbidity in this study. However, a 6.8% increase of neck dissection cases compared to thyroidectomy only cases between 2007 and 2011 suggests that the more patients underwent wider extent of neck surgery which generally relates to elevated postoperative morbidity.

It is uncertain why the proportion of neck dissections in thyroid cancer has been increasing in Korea. One possible reason is the development of diagnostic tools for LN metastasis. The US guided fine-needle aspiration and washout thyroglobulin level from suspicious lymph nodes has facilitated the preoperative evaluation of LN metastasis. Another possible reason is increased prophylactic central neck dissection. The *SND* increased from 6,194 cases to 15,394 cases absolutely, and from 21.6% to 25.24% relatively, for the 5 years of this study. The increase of *SND* might be associated with prophylactic central neck dissection.

Not only *SND* but also the proportion of other neck dissections (*MRND+RND+BND*) increased in this study from 11.1% to 14.46% over 5 years. Thus, a more likely reason is a true increase of thyroid cancer and LN metastasis related to radiation, diet, lifestyle, and environmental changes [3], [4]. The larger tumor size is associated with more frequent central and lateral node metastasis in papillary thyroid carcinoma [21], and there are many other reports that support the hypothesis that the frequency of large thyroid cancer is increasing [5]–[8]. Thus, these increases of large thyroid cancer might explain the increase of LN metastasis.

In other reports, papillary microcarcinoma (≤1 cm) accounts for 49% of the overall increase of thyroid cancer [22]. This increase of papillary microcarcinoma would explain the increase of *UT* contrast to reduction of *TT* in this study. Lobectomy should only be sufficient for low-risk unifocal intrathyroidal papillary carcinoma (<1 cm) in the absence of head and neck irradiation, or clinically and radiologically involved nodes according to ATA guidelines [14].

Mortality from thyroid cancer is not improved. In Korea, mortality from thyroid cancer is sustained at 0.6 deaths per 100,000 [12]. In the United States, thyroid cancer mortality is also reported stable at approximately 0.5 cases per 100,000 [22]. Despite the definite increase of papillary microcarcinoma by improved detection technique [8], the mortality as well as the morbidity of thyroid cancer has not improved.

The rate of thyroid surgeries in men was lower than in women, while thyroid cancer in men is increasing more rapidly than in women. This result is in accordance with the results for the rate of thyroid cancer registered in the National Cancer Center of Korea [13]. However, it is the opposite of previous reports from other countries, where the rate in women increased more than in men [5], [23]. The proportion of thyroidectomy without neck dissection (*UT+TT*) was not different between male (59.72%) and female (60.67%) patients, clinically. And other proportions of surgeries between men and women were similar clinically. These results are in agreement with other reports [24].

There was less than one year of differences of mean ages between male and female receiving different types of surgeries (Table 3). Although the differences were statistically significant, these differences were too small to have meaning clinically. We find that patients who underwent *UT* were younger than those receiving other surgeries. We could not find any references on this. However, we presume that *UT* patients had their cancers detected at an earlier stage and younger age than patients receiving other surgeries, or that *UT* was performed in relatively young patients by the surgeon, considering the prognosis.

## Conclusions

The number of thyroid surgeries is increasing. The proportion of neck dissections in thyroid surgeries also showed an increasing trend in Korea, despite the rate of thyroid cancer increasing and the more cancers are detected at that earlier stage.
